# Influence of perceived and actual neighbourhood disorder on common mental illness

**DOI:** 10.1007/s00127-013-0813-9

**Published:** 2014-01-01

**Authors:** C. Polling, M. Khondoker, SELCoH study team, S. L. Hatch, M. Hotopf

**Affiliations:** 1 Academic Department Psychological Medicine, Institute of Psychiatry, King’s College London, 10 Cutcombe Road, London, SE5 9RJ UK; 2 Department of Biostatistics, Institute of Psychiatry and NIHR Biomedical Research Centre for Mental Health at the South London and Maudsley NHS Foundation Trust, King’s College London, Box P020, De Crespigny Park, London, SE5 8AF UK

**Keywords:** Neighbourhood, Common mental disorder, Social disorder, Crime

## Abstract

**Purpose:**

Fear of crime and perceived neighbourhood disorder have been linked to common mental illness (CMI). However, few UK studies have also considered the experience of crime at the individual and neighbourhood level. This study aims to identify individual and local area factors associated with increased perceived neighbourhood disorder and test associations between CMI and individuals’ perceptions of disorder in their neighbourhoods, personal experiences of crime and neighbourhood crime rates.

**Methods:**

A cross-sectional survey was conducted of 1,698 adults living in 1,075 households in Lambeth and Southwark, London. CMI was assessed using the Revised Clinical Interview Schedule. Data were analysed using multilevel logistic regression with neighbourhood defined as lower super output area.

**Results:**

Individuals who reported neighbourhood disorder were more likely to suffer CMI (OR 2.12) as were those with individual experience of crime. These effects remained significant when individual characteristics were controlled for. While 14 % of the variance in perceived neighbourhood disorder occurred at the neighbourhood level, there was no significant variance at this level for CMI.

**Conclusions:**

Perceived neighbourhood disorder is more common in income-deprived areas and individuals who are unemployed. Worry about one’s local area and individual experience of crime are strongly and independently associated with CMI, but neighbourhood crime rates do not appear to impact on mental health.

## Introduction

There is increasing interest in the role of place in influencing a variety of health outcomes and in explaining health inequalities [1]. Within mental health, spatial patterning has long been noted in the incidence of suicide [2] and psychosis [3], and more recently various neighbourhood-level exposures have been found to influence these outcomes [4, 5].

Common mental illnesses (CMI) (i.e. depression and anxiety disorders) [6] are major contributors to the burden of disease globally, particularly in high-income countries [7]. Research on the influence of place on CMI over the past decade has been mixed. The prevalence of these disorders show stark social inequalities, with a greater proportion of those on lower incomes, the unemployed and those with fewer educational opportunities being affected [8]. The prevalence of these factors is differentially distributed across communities, with deprived neighbourhoods by definition having higher concentrations of impoverished and economically inactive individuals [9].

It is less clear whether the risk of CMI is also affected by social processes occurring at a neighbourhood level. Research in this area has been mixed, with two reviews finding some evidence, predominantly from the USA, of a link between deprivation and other neighbourhood problems and the prevalence of CMI that persists when individual factors are controlled for [1, 10]. The majority of the studies included in both these reviews were performed in urban areas, although some studies that have also included rural areas found no difference in CMI prevalence between the settings [11, 12]. Evidence from national UK samples has found relatively little variation in the prevalence of CMI between neighbourhoods which has generally been accounted for by individual and household factors [12–15]. Small neighbourhood-level effects have been found in female, non-white and lower educated sub-groups over and above individual risk factors [14, 16].

Much research looking for neighbourhood effects on CMI has used measures that are a summary of the characteristics of each area’s population [9]. For example, in the studies discussed above, area deprivation was generally characterised by measures constructed from summary statistics for the population including average income and rates of unemployment. When measured in this way, such area effects are likely to be difficult to separate from their analogous individual variable, particularly where the neighbourhoods studied are small and homogenous [13].

To find true neighbourhood effects separate from individual characteristics, it may be more fruitful to explore aspects of neighbourhood physical and social environment [9]. These have been less frequently investigated, not least because comparable objective measures of context for multiple small areas are much less readily available than summary statistics describing populations. Levels of disorder can be conceptualised as an aspect of both neighbourhood social environment, where levels of crime and anti-social behaviour influence feelings of safety and social connectedness, and the physical environment which may be degraded by graffiti and vandalism [9]. The potential for these factors to influence mental health has been reflected in the UK policy with measures of disorder included in national measures of well-being [17] and population mental health used to evaluate neighbourhood regeneration policies [18].

A review of the influence of neighbourhood characteristics on depression found some evidence supporting a harmful effect of neighbourhood disorder, although most of the studies included relied on respondent perception alone to measure disorder [10]. Ross and Mirowsky’s [11] work in Illinois suggested that the effect of neighbourhood deprivation on mental health was mediated by neighbourhood disorder. Work on fear of crime has also shown a link between individuals’ concerns about their local area and various worsened health outcomes including mental health [19]. Studies considering the effect of an unfavourable physical environment on mental health have hypothesised that this may act as a direct stressor that increases individuals’ vulnerability to anxiety and depression [20]. Meanwhile, work using the Whitehall II study data has suggested that worry about disorder in the local area has the effect of limiting involvement in social and physical activities. Such activities in turn may enhance well-being and provide a buffer against CMI [19]. Whitely and Prince found a similar effect of fear of crime in their qualitative work in inner city London [21].

In this study we used a broad definition of neighbourhood disorder which encompasses both physical and social aspects. Unlike many previous studies, we have included both a measure of individual’s’ perception of disorder in the local area and local crime rates as a more objective proxy for disorder as well as individual’s experience of victimisation. We examine experience of neighbourhood disorder and of CMI in an area of inner South London which is diverse both in terms of ethnicity and levels of neighbourhood deprivation and in which rates of CMI are high and disorder is a significant concern.

This study examines the association between experience of neighbourhood disorder and CMI. We first aim to examine the relationship between perceived neighbourhood disorder and individual demographics and experience of crime as well as area-level factors. We hypothesise that perceived neighbourhood disorder will be clustered by neighbourhood and be higher in areas with higher crime rates and amongst those with an individual experience of crime. We then test the hypotheses that CMI is clustered by neighbourhood and that (1) individual perception of neighbourhood disorder, (2) personal experience of violent victimisation and (3) higher neighbourhood crime rates are associated with higher prevalence of CMI.

## Method

Lambeth and Southwark are neighbouring boroughs in inner South London with a combined population of approximately 590,000. This population is ethnically diverse with over a third of residents belonging to black and minority ethnic groups and a similar proportion born outside of the UK [22]. Overall, the area is significantly more deprived than the national average with over 90 % of neighbourhoods studied scoring above the national median for deprivation on the Index of Multiple Deprivation 2010 (IMD). However, it also contains areas of significant wealth including some of the richest neighbourhoods in the UK [23]. The overall crime rate of 125/1,000 population for the two boroughs is well above the national average of 74/1,000 [22] and nearly 50 % of neighbourhoods studied were in the top quintile for the crime domain of the IMD.

### Study design and participants

The South East London Community Health (SELCoH) study surveyed 1,698 individuals in 1,075 randomly selected households within the London boroughs of Lambeth and Southwark between 2008 and 2010. Face-to-face interviews were carried out in participants’ homes by trained interviewers using a computer-assisted schedule. The survey collected data on psychiatric and physical morbidity, health behaviours and health service use as well as socio-demographics, psychosocial factors and neighbourhood characteristics. Full details of study design, participants, procedures and measures used have been published elsewhere [24].

### Measures

#### Exposures

Perceived neighbourhood disorder was determined from four questions: “Thinking of the area you live in, how much of a problem is each of the following?” asked regarding (1) vandalism/graffiti, (2) crime, (3) safety and (4) rubbish/litter. Responses were scored on a Likert scale as ‘Not a problem ‘(0), ‘Minor’ (1), ‘Somewhat serious’ (2) and ‘Very serious’ (3). Total score when all four questions were combined was not normally distributed and so a binary variable was created by splitting the highest rating given on any question into none/minor (low perceived disorder) and somewhat/very serious (high perceived disorder).

Individual experience of crime was defined by three variables. Participants were coded as having been victimised if they answered yes to any of the following: (1) “Have you ever been attacked, mugged, robbed or been the victim of a serious crime?”, (2) “Has anyone ever injured you with a weapon—gun, knife, stick, etc.?” or (3) “Has anyone ever hit you, bit you, slapped you, kicked you or forced you to have sex against your wishes?”. Participants were coded as having witnessed violence if they answered yes to “Have you ever seen something violent happen to someone (e.g. attacked or beaten) or seen someone killed?”. Participants were also asked whether the same events had happened to them in the past year.

#### Outcome

Presence of CMI was assessed using the Revised Clinical Interview Schedule (CIS-R), a semi-structured interview covering non-psychotic symptoms [25]. The conventional cutoff of a total score of 12 was used to define cases.

#### Potential confounders

Age, sex, ethnicity, education and occupation were included as individual-level variables, which were shown in previous research to be associated with CMI and fear of crime and hence potential confounders of the relationship between the two. Occupation was categorised using the Registrar General’s Classification of social class [26] condensed into two groups, manual or non-manual, for respondents currently in work. Participants who were retired, sick, disabled, students or caring for children were classified as economically inactive, while those seeking work were separately classified as unemployed. Household income (as well as occupation and education) was included as an indicator of an individual’s deprivation to allow the effect of this to be distinguished from neighbourhood-level deprivation.

#### Residential mobility

Having recently moved into a neighbourhood may be associated with different rates of survey participation, different perceptions of the neighbourhood and different experiences of violence (for example, people may have moved seeking out a safer neighbourhood). Participants were asked whether they had been at their current address for more or less than 2 years.

### Spatial scale

Full postcode data were available for each household and used to allocate them to lower layer super output areas (LSOAs) and Census Area Statistics wards (wards) using the Office of National Statistics Postcode Directory [27]. Analyses were performed using LSOA as a proxy for neighbourhood. LSOAs are statistical geographic units used by the Office of National Statistics (ONS) for reporting census data. While they cannot be considered synonymous with neighbourhoods, this level of geography has the benefit of a more local scale than wards, as LSOAs have a mean population of 1,500 compared to ward populations of 10,000–15,000 in Lambeth and Southwark [28]. They are the standard unit used for publishing ONS neighbourhood statistics including the IMD.

### Neighbourhood-level variables

The ONS IMD 2010 was used to define neighbourhood levels of deprivation. IMD is the government’s official measure of deprivation at the small area level and scores are published for every LSOA in England [29]. The IMD 2010 is based on data from 2008 for 38 indicators grouped into seven domains and is designed to capture multiple aspects of deprivation. Scores do not indicate absolute differences between areas, but are ranked to allow relative deprivation between areas to be explored.

Total IMD contains a health sub-domain which includes measures that aim at estimating local rates of mental disorder, so for this analysis the income and crime sub-domains were used on their own as well as overall IMD rank. The income sub-domain is based on a count of the proportion of an LSOA’s population who are income deprived, indicated by the receipt of means-tested benefits. The crime sub-domain is based on the police-recorded rates of (1) violent crime, (2) burglary (3), theft and (4) criminal damage, standardised to the resident and workplace population of the LSOA [30].

Although the boroughs of Lambeth and Southwark have areas of low deprivation compared to England as a whole, the majority of both boroughs are more deprived than the national average. To allow for useful comparisons of relative deprivation locally to be made, the LSOAs in Lambeth and Southwark were grouped into local tertiles for analyses.

### Statistical methods

Analyses were performed in STATA version 11 [31]. Initial descriptive analyses were performed using survey commands (svy) to account for clustering by household due to the study design and were weighted for non-response within households. The process for calculating the weights has been published elsewhere [24].

### Multilevel models

Random effects logistic regression analyses were performed for the binary outcomes: (1) high perceived neighbourhood disorder and (2) CMI. A three-level random intercept logistic model was used to account for the hierarchical nature of the data considering individuals as level 1, households as level 2 and neighbourhood as level 3. Analyses used the STATA command GLLAMM version 2.3.15 [32] using a logit link function and binomial family for the distribution of outcomes. Simple logistic models considering one covariate at a time were used to estimate unadjusted odds for individual and neighbourhood-level variables. Model 1 in each case mutually controlled for individual sex, age, ethnicity, occupational class and education and household income. Model 2 added variables measuring individual experience of crime. Additional models were then produced adding each neighbourhood-level variable individually to Model 2.

The proportion of residual variance occurring at each level of the model was assessed first using a null model with no covariates controlled for and again for Model 2. To estimate the proportion of the residual variance occurring at each level, an underlying linear random intercept model for the latent propensity to the binary outcome, defined by a threshold, was assumed. Hence the residual variance at the individual level was assumed to follow a standard logistic distribution and so fixed at the standard logistic variance of 3.29 (π^2^/3) (see Snijders and Bosker [33] for further discussion of estimates of variance from multilevel logistic models). Variance partition coefficients were calculated for each level by dividing the residual variance at the level by the total residual variance. Two- and three-level models for each set of covariates modelled were also compared using likelihood ratio tests.

## Results

### Participation rates

At least one person was interviewed in 51.9 % of eligible households contacted. Within participating households, 71.9 % of eligible adults participated. The sample was similar to the 2011 census sample in terms of demographics and socio-economic indicators, with the exception of the sample being slightly younger and having more students among the economically inactive [34]. There were participants located in 322 of the 342 LSOAs in Lambeth and Southwark with a range of 1–18 participants per LSOA. There were participants in all 42 wards within the two boroughs with a range of 19–68 per ward.

The prevalence of CMI amongst all participants was 24.2 %. Personal experience of crime was common with 51.7 % of the sample having been victimised at some time in their lives and 41.5 % having witnessed violence. Table 1 shows the demographic characteristics and individual experience of crime in the sample.

**Table 1:** Perceived neighbourhood disorder and common mental illness by demographic characteristics in the SELCoH sample

	*n*	High perceived disorder, *n* (%^a^)	Case on CIS-R *n* (%^a^)
Total	1,698	626 (37.6 %)	396 (24.2 %)
Sex		*n* = 1,663	*n* = 1,692
Male	739	255 (34.7 %)	131 (18.0 %)
Female	959	371 (39.1 %)	265 (27.3 %)
Age group		*n* = 1,663	*n* = 1,692
16–24	356	158 (44.7 %)	84 (25.1 %)
25–34	404	137 (35.1 %)	88 (22.8 %)
35–44	336	122 (37 %)	77 (24.3 %)
45–54	264	101 (40.5 %)	75 (30.1 %)
55–64	163	51 (33.3 %)	41 (25.4 %)
65+	175	57 (34.1 %)	31 (18.3 %)
Ethnicity		*n* = 1,661	*n* = 1,690
White	1,051	402 (39.3 %)	250 (24.4 %)
Black Caribbean	143	55 (38.0 %)	41 (31.0 %)
Black African	234	80 (34.5 %)	44 (19.5 %)
Asian	63	20 (33.1 %)	14 (24.9 %)
Other	205	68 (32.7 %)	46 (23.0 %)
Annual household income		*n* = 1,640	*n* = 1,669
<5 k	139	53 (40.2 %)	60 (42.2 %)
£5–12 k	212	93 (43.5 %)	58 (26.6 %)
£12–20 k	203	83 (43.4 %)	56 (29.0 %)
£20–31 k	179	69 (38.6 %)	40 (23.2 %)
>£31 k	703	223 (32.2 %)	129 (18.8 %)
Don’t know	239	96 (39.3 %)	50 (22.7 %)
Highest Ed qualification		*n* = 1,644	*n* = 1,673
None	228	88 (40.2 %)	61 (25.7 %)
GCSE	332	144 (43.1 %)	100 (30.5 %)
A-Level	426	176 (42.8 %)	102 (25.6 %)
Degree or above	693	211 (30.8 %)	127 (19.2 %)
Occupational class		*n* = 1,654	*n* = 1,683
Non-manual	694	231 (34.1 %)	133 (19.6 %)
Manual	231	82 (36.2 %)	46 (21.2 %)
Student	243	104 (43.3 %)	46 (20.1 %)
Unemployed	170	72 (44.9 %)	58 (35.5 %)
Ec inactive	351	132 (37.8 %)	111 (30.1 %)
Ever victimised		*n* = 1,662	*n* = 1,676
No	794	252 (32.4 %)	122 (15.7 %)
Yes	888	374 (42.6 %)	267 (31.8 %)
Ever witnessed violence		*n* = 1,662	*n* = 1,676
No	1,535	551 (36.5 %)	344 (23.3 %)
Yes	147	75 (51.6 %)	45 (32.7 %)

*CIS-R* revised clinical interview schedule, *SELCoH* South East London Community Health Survey

^a^Weighted percentages

### Perceived neighbourhood disorder

At least one somewhat or very serious disorder problem was reported by 37.6 % of participants. The most commonly reported problem was crime (27.4 %) followed by safety concerns (16.1 %), litter (15.8 %) and vandalism (10.4 %). The percentages reporting high levels of disorder in different demographic groups are shown in Table 1.

Perception of neighbourhood disorder was greatest amongst 16- to 24-year-olds, students and the unemployed (Table 2). When individual demographic factors were adjusted for simultaneously, perceived neighbourhood disorder was significantly lower in older people. Those from the white ethnic group were more likely to report neighbourhood disorder than other ethnic groups, with the effect reaching statistical significance for black African and “other” ethnic groups. Having a personal experience of crime was associated with increased perceived disorder and adjusting for this (Model 2) revealed higher perceived disorder in women which had not been statistically significant in earlier models.

**Table 2:** Associations between individual factors and perceived neighbourhood disorder (three-level logistic regression)

	Odds ratio for high perceived neighbourhood disorder (95 % CI)					
	Unadjusted OR		Model 1^a^		Model 2^b^	
Sex						
Male	1.00		1.00		1.00	
Female	1.26	(0.95–1.68)	1.20	(0.90–1.61)	1.49	(1.08–2.05)*
Age						
16–24	1.00		1.00		1.00	
25–34	0.62	(0.39–0.98)*	0.74	(0.44–1.24)	0.75	(0.44–1.28)
35–44	0.63	(0.39–1.02)	0.79	(0.47–1.34)	0.81	(0.46–1.40)
45–54	0.76	(0.43–1.32)	0.76	(0.42–1.37)	0.78	(0.42–1.43)
55–64	0.47	(0.26–0.85)*	0.46	(0.24–0.88)*	0.47	(0.24–0.92)*
65+	0.57	(0.32–1.01)	0.42	(0.19–0.91)*	0.51	(0.23–1.13)
Ethnicity						
White	1.00		1.00		1.00	
Black Caribbean	0.89	(0.50–1.61)	0.65	(0.36–1.20)	0.65	(0.35–1.21)
Black African	0.66	(0.39–1.11)	0.55	(0.32–0.93)*	0.57	(0.33–0.99)*
Asian	0.63	(0.29–1.41)	0.58	(0.27–1.24)	0.68	(0.30–1.53)
Other	0.64	(0.38–1.07)	0.52	(0.30–0.88)*	0.52	(0.30–0.91)*
Occupation						
Non-manual	1.00		1.00		1.00	
Manual	0.95	(0.58–1.54)	0.80	(0.48–1.34)	0.79	(0.47–1.32)
Student	1.66	(1.05–2.63)*	1.10	(0.64–1.88)	1.07	(0.61–1.87)
Unemployed	1.71	(1.01–2.89)*	1.24	(0.69–2.24)	1.18	(0.64–2.17)
Ec Inactive	1.23	(0.82–1.85)	1.09	(0.63–1.91)	1.04	(0.58–1.84)
Household income						
<5 k	1.00		1.00		1.00	
£5–12 k	1.34	(0.69–2.62)	1.49	(0.76–2.91)	1.74	(0.87–3.48)
£12–20 k	1.11	(0.56–2.19)	1.27	(0.64–2.55)	1.46	(0.71–3.00)
£20–31 k	0.91	(0.47–1.75)	1.10	(0.56–2.19)	1.18	(0.57–2.42)
>£31 k	0.73	(0.42–1.25)	0.88	(0.47–1.68)	0.97	(0.50–1.87)
Don’t know	0.98	(0.52–1.85)	0.97	(0.51–1.87)	1.12	(0.58–2.19)
Education						
None	1.00		1.00		1.00	
GCSE	1.10	(0.64–1.91)	1.02	(0.57–1.86)	1.04	(0.56–1.94)
A-Level	1.02	(0.61–1.70)	0.92	(0.51–1.66)	0.91	(0.50–1.65)
Degree or above	0.62	(0.37–1.02)	0.60	(0.32–1.12)	0.61	(0.32–1.14)
Ever victimised						
No	1.00				1.00	
Yes	1.80	(1.31–2.49)***			1.49	(1.05–2.10)*
Ever witnessed violence						
No	1.00				1.00	
Yes	2.12	(1.54–2.92)***			2.02	(1.43–2.87)***

* *p* < 0.05, ** *p* < 0.01, *** *p* < 0.001

^a^Model controlled for sex, age, ethnicity, household income, education and occupation

^b^Controlled for sex, age, ethnicity, household income, education, occupation, victimisation and witnessing violence

Participants whose neighbourhoods were characterised by higher crime rates, greater income deprivation and higher total deprivation tended to have increased concern about disorder. The odds ratios presented in Table 3 are for each tertile of deprivation with, for example, participants from neighbourhoods with the highest total deprivation having three times the odds of reporting neighbourhood disorder compared to those in the least deprived tertile. This effect remained when sex, age, ethnicity, household income, education, occupation, victimisation and witnessing violence were controlled for (Model 2). The effect of neighbourhood crime rates on perception of neighbourhood disorder was lower than that of income deprivation and total deprivation, and became non-significant when income deprivation was controlled for simultaneously.

**Table 3:** Associations between neighbourhood factors and perceived neighbourhood disorder (three-level logistic regression)

	Odds ratio for high perceived neighbourhood disorder (95 % CI)			
	Unadjusted OR		Added to model 2^a^	
			Variables added singly	
IMD Crime domain				
1st tertile (least deprived)	1.00		1.00	
2nd tertile	1.30	(0.79–2.15)	1.29	(0.78–2.15)
3rd tertile (most deprived)	1.63	(1.03–2.58)*	1.65	(1.05–2.60)*
IMD Income domain				
1st tertile (least deprived)	1.00		1.00	
2nd tertile	1.73	(1.08–2.79)*	1.60	(0.98–2.60)
3rd tertile (most deprived)	2.57	(1.57–4.20)***	2.63	(1.57–4.42)***
Total IMD				
1st tertile (least deprived)	1.00		1.00	
2nd tertile	2.59	(1.60–4.19)***	2.44	(1.48–4.02)***
3rd tertile (most deprived)	3.05	(1.85–5.03)***	3.16	(1.88–5.29)***
	Odds ratio for high perceived neighbourhood disorder (95 % CI)			
	Unadjusted OR		Added to model 2^a^	
			Crime and income added simultaneously	
IMD Crime domain				
1st tertile (least deprived)	1.00		1.00	
2nd tertile	1.26	(0.78–2.04)	1.26	(0.77–2.07)
3rd tertile (most deprived)	1.44	(0.91–2.28)	1.48	(0.94–2.33)
IMD Income domain				
1st tertile (least deprived)	1.00		1.00	
2nd tertile	1.63	(1.00–2.65)	1.49	(0.90–2.45)
3rd tertile (most deprived)	2.46	(1.50–4.01)***	2.49	(1.49–4.18)**

*IMD* index of multiple deprivation 2010

* *p* < 0.05, ** *p* < 0.01, *** *p* < 0.001

^a^Controlled for sex, age, ethnicity, household income, education, occupation, victimisation and witnessing violence

A null three-level model was used to estimate the proportion of variance in perceived neighbourhood disorder occurring at each level. Where neighbourhood was defined as LSOA, variance at the individual level was fixed at 3.29 which represented 53.9 % of the total variance, variance at the household level was 1.96 (SE 0.52), 32.1 % of total variance, and at the neighbourhood level 0.85 (SE 0.25) and 14.0 % of total variance. A likelihood ratio test comparing the three-level model with a two-level model showed that the three-level model better accounted for the data (*χ* ^2^ = 14.6 *p* < 0.0005). These proportions remained similar when individual factors were controlled for (variance at neighbourhood level 0.76 (SE 0.26), 12.6 % of total variance). The proportion of variance at the neighbourhood level fell to 0.52 (SE 0.23), 9.0 % of total variance, but remained significant when income and crime deprivation were controlled for, suggesting that around a quarter of the variance within neighbourhoods is accounted for by these deprivation indices.

### Common mental illness

Perceived neighbourhood disorder was associated with the presence of CMI with an unadjusted OR of 2.12 in the base model (Table 4). This effect was partially attenuated by controlling for individual demographic factors and individual experience of crime, but remained sizeable and significant (OR 1.55 *p* = 0.007) when these factors were controlled for. Neighbourhood crime rates, income deprivation or total deprivation was not associated with CMI, before or after controlling for individual factors. However, individual experience of crime was strongly associated with the presence of CMI.

**Table 4:** Associations between perceived neighbourhood disorder, individual and neighbourhood factors and common mental illness (three-level logistic regression)

	Odds ratio for being a case on CIS-R (95 % CI)					
	Unadjusted OR		Model 1^a^		Model 2^b^	
Individual variables						
Neighbourhood disorder						
Low	1.00		1.00		1.00	
High	2.12	(1.54–2.91)***	1.84	(1.33–2.55)***	1.55	(1.13–2.13)**
Ever victimised						
No	1.00				1.00	
Yes	3.26	(2.24–4.72)***			2.58	(1.77–3.77)***
Ever witness violence						
No	1.00				1.00	
Yes	2.40	(1.71–3.37)***			2.06	(1.42–2.99)***
Neighbourhood variables					Variables added singly to Model 2:	
IMD Crime domain						
1st tertile (least deprived)	1.00				1.00	
2nd tertile	1.23	(0.81–1.87)			1.17	(0.79–1.73)
3rd tertile (most deprived)	1.02	(0.66–1.57)			0.96	(0.63–1.46)
IMD Income domain						
1st tertile (least deprived)	1.00				1.00	
2nd tertile	1.49	(0.98–2.25)			1.18	(0.80–1.74)
3rd tertile (most deprived)	1.46	(0.96–2.21)			1.14	(0.74–1.76)
Total IMD						
1st tertile (least deprived)	1.00				1.00	
2nd tertile	1.68	(1.12–2.52)			1.25	(0.85–1.84)
3rd tertile (most deprived)	1.55	(1.02–2.37)*			1.27	(0.83–1.95)

*CIS-R* revised clinical interview schedule, *IMD* index of multiple deprivation 2010

* *p* < 0.05, ** *p* < 0.01, *** *p* < 0.001

^a^Controlled for sex, age, ethnicity, household income, education and occupation

^b^Controlled for sex, age, ethnicity, household income, education, occupation, victimisation and witnessing violence

Estimates of the proportion of variance at each level from the null model showed significant variance at the individual level (fixed at 3.29, 56.8 % of total variance) and household level [2.50 (SE 0.58), 42 % of total variance], but no significant variance at the neighbourhood level (variance <0.001). A likelihood ratio test comparing the three-level model with a two-level model showed no additional benefit to including a third level (*χ* ^2^ = 0.63 *p* = 0.43). This remained the case when models controlling for individual and neighbourhood-level variables were considered.

The above multilevel models were repeated with level 2 defined as wards. The resultant odds ratios and confidence intervals were very similar in all models despite the definition of neighbourhood being much larger (data not shown here).

### Residential mobility

There were 588 participants (30.0 %, weighted for non-response) who had been living at their current address for <2 years. Those who had moved in the past 2 years were significantly younger, more likely to be in higher income and better educated groups and more likely to be economically active in non-manual work or be students than those who had not (data not shown here). Those who had moved in the last 2 years were no more likely to score as cases on CIS-R than those who had not (OR 0.87, 95 % CI 0.69–1.11).

Without neighbourhood-level data on residential mobility, it is not possible to say whether areas of greater mobility were also more disordered. However, individuals who had moved in the past 2 years were less likely to report neighbourhood problems than those who had not (OR 0.62, 95 % CI 0.50–0.77). Those who had ever been victimised were no more likely to have moved in the past 2 years than those who had not. Those who had been victimised in the past year were more likely to have moved in the past 2 years (OR 1.90 95 % CI 1.31–2.76); however, this association was confounded by the youthful demographic of the more mobile population and was reduced and no longer significant when age was controlled for (OR 1.37 95 % CI 0.92–2.03). Overall, there was not evidence that residential mobility might act as a confounder of associations between perceived neighbourhood disorder, victimisation and CMI.

### Sensitivity analyses

The sample included some LSOAs which contained only small numbers of individuals for analysis. The sensitivity of the results to the inclusion of these LSOAs was tested by repeating the analyses excluding LSOAs which contained fewer than five individuals. This reduced the sample to 1,175 individuals in 714 households and 152 LSOAs. A summary of the results is given below; full tables are not shown here for space reasons.

In the analyses with perceived neighbourhood disorder as the outcome, the associations between individual factors and increased perceived disorder all remained in the same direction, although the trend to decreased perceived disorder in non-white ethnic groups and increased perceived disorder in students and the unemployed were no longer significant at the 5 % level. For neighbourhood-level factors, all the effects reported remained significant when LSOAs with few participants were excluded and in most cases effect sizes and significance were increased.

Analyses with CMI as the outcome showed very little change when LSOAs with few participants were excluded. All the effects noted in the main analysis remained with similar effect sizes and significance.

With all the above analyses, the proportions of the variance reported at each level remained similar when LSOAs with few participants were excluded, with a small reduction in the proportion of variance at the household level and corresponding increase in variance at the individual level. For example in the null three-level model with CMI as an outcome reported above, household variance reduced to 2.03 (SE 0.56), falling from 43.2 % of total variance to 38.2 %, while individual variance increased from 56.8 % of total variance to 61.8 % (actual variance fixed at 3.29 in both models) and neighbourhood-level variance remained <0.0001 and non-significant.

Given that household-level variances remained surprisingly high in all our models compared to those reported in the literature, the null model with CMI as an outcome was also repeated excluding households with only one respondent. This produced a further reduction in household-level variance to 1.36 (SE 0.49), 29.1 % of total variance, with an increase in the proportion of individual-level variance to 70.5 % and a very small and non-significant increase in neighbourhood-level variance to 0.02 (SE0.24), 0.4 % of total variance.

## Discussion

### Experience of neighbourhood disorder

Concern about neighbourhood disorder and in particular crime was common in our sample compared to national figures [35]. This reflects the recorded crime statistics for Lambeth and Southwark boroughs, which both rank highly in levels of crime and anti-social behaviour nationally, as well as a wider population perception of them as high crime areas [36], and supports the use of crime rates as our objective measure of disorder. The individual-level risk factors examined also suggest that perception of neighbourhood disorder is related to objective experience, with victimisation or witnessing violent crime being the strongest individual-level associations.

The 16- to 24-year age group had the greatest concern about disorder, while the over 65 age group had fewer concerns. Whilst lower concern about crime in the elderly may appear counterintuitive, it is in keeping with national samples [35]. These differences are partly explained by the association seen between personal experience of crime and perceived neighbourhood disorder. In our sample 14 % of individuals aged 16–24 reported having been victimised in the past year and 18 % reported having witnessed violence in that time, compared with 4 and 5 %, respectively, in other age groups. Young people without direct experience of crime may nonetheless have increased concerns due to their realistic understanding that they are at much higher risk of victimisation than the population as a whole.

In contrast to national samples [35], univariate analyses did not show a significantly higher concern about neighbourhood disorder amongst women. However, this expected effect was revealed in models controlling for experience of crime. This indicates that the effect of gender was being suppressed by the impact of individual experience of crime. Men more commonly reported experiencing victimisation and witnessing violence in this sample and this may be acting to increase their prevalence of concern about disorder.

Examination of the variance in perceived disorder indicated that there was clustering of high perceived neighbourhood disorder by LSOA, suggesting that where people live makes a significant contribution to perception in addition to the effect of individual characteristics. Living in a high crime neighbourhood was associated with an increase in perceived disorder of a similar magnitude to that associated with individual experience of crime. However, the effect of deprivation was larger and area-level income deprivation appears to account for the effect of neighbourhood crime rates when both are controlled for. This might be taken as an indication that individual perceptions of neighbourhoods as disordered and unsafe are more related to visible physical disorder associated with deprivation than specific incidents of crime. Furthermore, crime and income deprivation together account for only about a quarter of the variance at neighbourhood level, suggesting that other area-level factors also play an important role.

### Common mental illness

We found a strong association between perceived neighbourhood disorder and CMI. This association was not simply an effect of confounding by demographic and socio-economic factors. Victimisation and witnessing violence were both also strongly linked with CMI, but these factors only accounted for part of the effect of perception of neighbourhood disorder on CMI.

The relationship between perceived neighbourhood disorder and CMI is likely to be a complicated one. Feeling unsafe and under threat in one’s local area could potentially act as a direct stressor on individuals, especially those in groups whose daily activities are most restricted to their immediate locality, such as the unemployed [11]. These groups are already at increased risk of CMI. Perhaps more significantly, such perceived disorder reduces individuals’ ability to take part in social and physical activities outside the home that might be important in protection and recovery from such illnesses [19, 21]. This study demonstrates that concerns about disorder in the neighbourhood are concentrated in areas where more income-deprived people live and so have the potential to be exacerbating inequalities in mental health outcomes.

Although individuals’ perception of their neighbourhood appears to be linked to mental health, these data did not suggest that the place where people lived had an effect. In common with other UK studies [15, 16], we found no significant variance in CMI at neighbourhood level, despite this study using a smaller unit of neighbourhood and a more robust measure of CMI than most previous studies.

We did not find an independent effect of neighbourhood crime rates or deprivation on CMI, in contrast to the association with subjective perception. This may highlight that police-recorded crime rates are an imperfect measure of the true experience of neighbourhood disorder as they reflect a relatively small proportion of total crime [39] and may be more likely to miss crimes in deprived areas due to underreporting. However, crime is not an environmental factor that is necessarily experienced by the whole population: the impact of a crime within a neighbourhood may be profound for some individuals directly experiencing it, but have little or no direct impact on the majority.

These findings suggest that reducing perceived neighbourhood disorder is a worthwhile target for improving population mental health. However, the most useful interventions may be those targeted at specific population groups, for example young people and those who have been victims or witnesses to crime, rather than those targeted at a neighbourhood as a whole. Measures of neighbourhood deprivation may be more useful than crime rates in identifying geographical areas in which to target these higher risk groups.

### Strengths and limitations

This study adds to the existing literature on the influence of neighbourhood disorder in a number of ways. It investigated an inner city population which is diverse, especially in terms of ethnicity and socio-economic status, and subject to high levels of deprivation and neighbourhood disorder.

Using cross-sectional data, it is not possible to ascertain the direction of causation for the association between perceived neighbourhood disorder and CMI. Information biases are important; participants who were cases on the CIS-R were asked about their neighbourhood at a time when they reported a recent experience of anxiety or depression symptoms and these are likely to colour their perception of their local area. However, the difference in the spatial patterning of perceived disorder from that of CMI indicates that these responses did not simply measure the same thing. Furthermore, perception of disorder was associated with objective measures of neighbourhood problems, particularly deprivation, independent of individual characteristics, while CMI was not.

A limitation of this study is the relatively low household participation rate of 51.9 %. This is in part a reflection of the difficulty of conducting surveys in deprived inner city environments, and the participation rates reported are relatively high compared with recent surveys in similar areas [24]. It is known that non-participation in surveys is strongly associated with the presence of mental disorder, so it may be that the rates of CMI reported in this sample, although high in comparison to national UK samples [24], are an underestimate. Work looking at the effect of non-participation suggests that while it is a significant problem for prevalence studies, it only modestly reduces associations between exposures and outcome [37]; however it may have reduced the effect sizes found in this study.

Individuals who move home frequently might be expected to be less likely to participate in surveys and hence be underrepresented in this study. In our sample 30 % of individuals had moved in the past 2 years. Greater London Authority (GLA) figures for 2008/09 estimate that the proportion of individuals in Lambeth who had been living at a previous address 1 year before was 17.0 %, while in Southwark it was 14.9 % [38]. This suggests that the sample contains approximately the expected numbers of residentially mobile individuals. The lack of association between residential mobility and victimisation, perceived disorder or CMI suggests it is unlikely that residential mobility confounds the associations reported.

### Choice of exposures

Much work on neighbourhood effects has used measures which summarise population characteristics and so are difficult to interpret when individual factors are also controlled for [9]. The use of crime rates is a step towards considering a neighbourhood’s environment separate from its population. It is possible that a stronger relationship with area-level variables was not observed because most of the areas within the study had levels of crime and deprivation that are high on a national comparison, limiting the variation between neighbourhoods and so reducing our ability to detect neighbourhood effects.

The measures of individuals’ experience of actual violent crime suggest that this is a strong influence on perception. The small numbers reporting experience of violence in the past year prevented the use of these variables in the main models, so those ever having experienced violence were used instead. The lack of information about the timing and location of reported experiences of violence means that individuals’ experience cannot be taken as a measure relating to their neighbourhood. However, the persistence of a strong association between perceived disorder and CMI despite inclusion of data about individual experience is something that has not been possible in much previous research linking fear of crime to CMI, and demonstrates that this relationship exists independently of actual experience of victimisation.

### Definition of neighbourhood

As with all research on neighbourhood effects, our definition of neighbourhood is imperfect and cannot be assumed to be the same area that people were thinking of when asked about perceived disorder. As a concept, neighbourhood is generally taken to refer to the shared space around clusters of residences that have similar attributes in terms of the individuals living there and the physical and social environment [40]. The definition of a specific neighbourhood is then likely to be dynamic and vary according to which attributes are of interest. Indeed, where individuals are asked about their neighbourhood, the area being described may well vary for every person asked. For the purposes of research, however, one set of boundaries must be imposed, raising the modifiable areal unit problem: that the results of analyses of local areas will vary according to the scale and the boundaries chosen [41]. This difficulty occurs where the boundaries imposed are arbitrary and lessened where there is a theoretical underpinning for the choice of neighbourhood used [42], although this will always involve a trade-off with pragmatic concerns.

To use secondary data as a measure of wider neighbourhood environment, we were constrained to using administrative boundaries in this study. The concentration of our sample in a relatively small geographical area allowed us to use LSOAs as our definition of neighbourhood. This definition has the benefit of being both smaller and more homogeneous than the electoral wards used in many previous studies [28] which may make inequalities between areas and area-level effects on perception of the social environment easier to detect [42]. We were also able to test a previous suggestion that the use of larger neighbourhood units could have masked underlying neighbourhood effects in some earlier, negative studies [13]. Our analysis was limited by the small numbers of individuals in some of the LSOAs. The sensitivity analyses excluding these LSOAs suggest that this did not affect the direction of associations seen, but that some of the neighbourhood-level effects may have been underestimated in our analyses because they could not be detected where there were only one or very few individuals in an LSOA.

The clustered sampling in this study allowed us to model household variance. Previous research has shown that it is important to consider the household level separately to the individual level when considering neighbourhood effects on mental health [15] to reduce the risk of attributing too much of the variance above individual level to the neighbourhood. Our ability to model household variance may have been limited by the fact that more than half our households only had one respondent. We found a residual variance at the household level that was significantly higher than in most previous studies, although when one-person households were excluded we found a very similar household variance to another recent UK study that used LSOA as its definition of neighbourhood [43]. This high household variance highlights that effects operating at the household level were particularly important in our study population, suggesting that responses to higher, area-level influences are similar for members of the same household but vary considerably between households.

The difficulties inherent in defining neighbourhood are exacerbated by the use of multilevel modelling techniques that treat individual neighbourhoods as independent and cannot easily account for the likelihood that geographically close neighbourhoods are more similar than those further apart. The finding that CMI was not spatially patterned within the sample is counterintuitive in many ways, and it is possible that geographical patterns exist in the data that could not be detected by the statistical methods used but may be found on a spatial statistical analysis [18].

## Conclusion

This study highlights that physical and social disorder within neighbourhoods has an important, but complicated relationship with CMI. Officially recorded crime rates appear to have a surprisingly modest association with individuals’ perception of neighbourhood disorder and little impact on mental health. At the same time individuals’ perception of their local neighbourhood and their own experience of violence have strong independent associations with CMI. These more subjective variables may capture aspects of the experience of living in disordered neighbourhoods that crime rates are unable to. Feeling unsafe and under threat in one’s local area disproportionately affects those already experiencing other forms of deprivation in their area and personally. Interventions aimed at reducing the impact of disordered neighbourhoods on mental health may help reduce inequalities in CMI by targeting both factors associated with increasing people’s perception of disorder and the impact of victimisation on individuals.

## Appendix

SELCoH Study Team: Matthew Hotopf, Stephani L Hatch, Souci Frissa, Maria Verdecchia, Robert Stewart, Nicola T Fear, Abraham Reichenberg, Craig Morgan, Bwalya Kankulu, Jennifer Clark, Billy Gazard, Robert Medcalf, Alistair Baile, Elizabeth Doherty, Deborah Bekele, Joshua Buckman, Grant Henderson, Max Henderson and Benedict Weobong.
